# Retinopathy of prematurity: it is time to take action

**Published:** 2017

**Authors:** Clare Gilbert, Hannah Blencowe

**Affiliations:** Professor of International Eye Health and Co-director: International Centre for Eye Health, London School of Hygiene & Tropical Medicine, London, UK.; Assistant Professor: London School of Hygiene & Tropical Medicine, UK.

**Figure:**
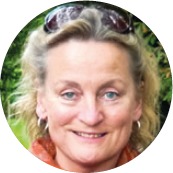
Clare Gilbert

**Figure:**
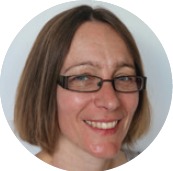
Hannah Blencowe

**Retinopathy of prematurity affects babies born preterm: before 37 weeks of gestation. Unless these babies are carefully managed, they can become visually impaired or blind. But there is hope: the condition can be prevented and treated.**

**Figure:**
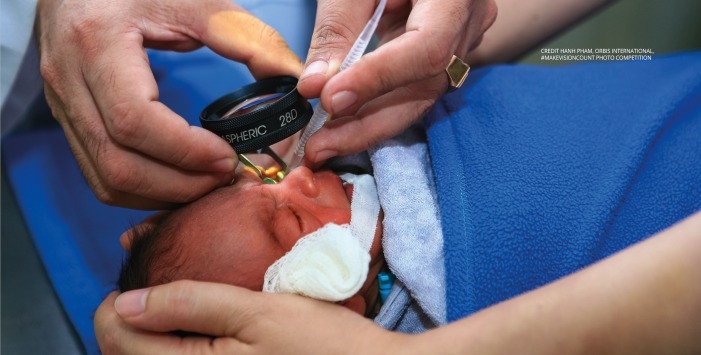
Preterm baby being screened for ROP using indirect ophthalmoscopy. VIETNAM

Every year, an estimated 15 million babies are born preterm (normal gestation is 37–42 weeks).^1^ Approximately 20,000 of these babies will become blind from retinopathy of prematurity (ROP) every year, and an additional 12,300 will be left with visual impairment.^2^

Countries with the highest number of preterm births are India, China, Nigeria, Pakistan and Indonesia. East Asia, South East Asia, and the Pacific are the regions with the highest number of preterm babies who survive, and the highest number who develop visual loss from ROP (Figure 1).^2^ However, all regions of the world are now affected.

For almost 80 years, it has been known that preterm infants can become blind from ROP: it was first described in the United States of America as retrolental fibroplasia. The main risk factors have also been known for a long time. Urgent laser treatment has now been shown to be effective, and screening and treatment programmes have reduced blindness in children from ROP in many high-income countries. So why is ROP an important cause of blindness in children in many low- and middle-income countries? There are four main reasons.

1. Increased services for sick and preterm infants mean that many more preterm babies are now surviving. Prematurity is responsible for 18% of under-5 mortality worldwide^3^, and governments have been motivated to address this by increasing the availability of neonatal services.
2. The quality of the neonatal care babies receive can be less than ideal in some areas, which increases the risk of the severe, sight-threatening stages of ROP.
3. Not all preterm infants at risk of ROP are screened, or screening is inadequate, and so babies requiring treatment are not identified.
4. Urgent laser treatment, which is highly effective in most cases, may not be delivered in time, or it may not be adequately delivered.

## Which babies are most at risk?

In the womb, the developing fetus is in a stable, warm, quiet, and dark environment, and is suspended in fluid and therefore able to move. Nutrients and oxygen are continuously supplied via the umbilical cord. Replicating this level of stability in babies who are ‘born too soon’ is a great challenge.

The following babies are at risk of ROP:

- Babies who are extremely premature, i.e., born more than 8 weeks early with a gestational age of less than 32 weeks. These babies are most at risk: the more preterm the baby, the greater the risk.
- Babies with a gestational age of 32–36 weeks (4–8 weeks premature), if they receive poor neonatal care.
- Babies who have a low birth weight (<1,500 g).
- Babies with a higher birthweight, if they receive poor neonatal care.
- Babies who are given too much oxygen and for too long (high blood oxygen levels damage the developing blood vessels in the retina).

The risk of ROP is increased by:

- Inadequate nutrition with poor weight gain during the first few weeks of life.
- Infection during the first few weeks.
- Anything that makes babies unstable: pain, poor temperature control and not keeping the baby comfortable and supported in the cot or incubator.

**Figure 1:**
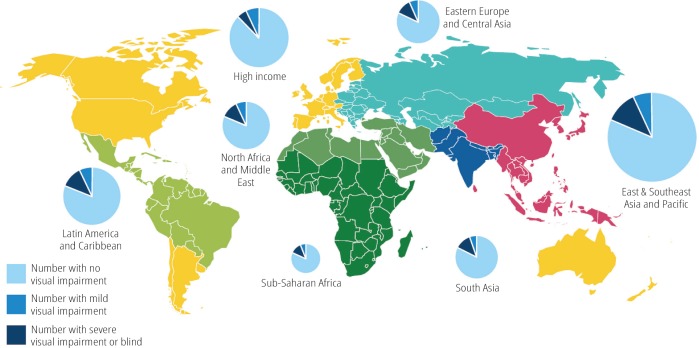
Number of preterm infants who survived in 2010 without visual impairment, with visual impairment, and blind. From Blencowe *et al.*^1^

Exposure to postnatal risk factors is higher in neonatal units where:

- Staff members are inadequately trained.
- There are too few staff members.
- There is inadequate equipment to deliver and monitor oxygen.
- Mothers are not encouraged to play a role in caring for and giving their babies breast milk.

## How can visual loss be prevented?

Premature birth is very difficult to predict or control, but good neonatal care, screening, and urgent laser treatment can reduce the number of infants who become blind or visually impaired.

The articles on pp. 50–54 explain how doctors and nurses can reduce the risk of ROP using the POINTS of Care system: controlling **pain,** careful use of **oxygen,** preventing **infection,** improving **nutrition** by offering babies breast milk, good **temperature** control and **supportive** practices to keep babies comfortable and stable, such as kangaroo care.

Screening for ROP is needed to detect babies who develop the serious, sight-threatening stages of ROP (pp. 57–58). Screening is usually conducted by an experienced ophthalmologist in the neonatal unit, using indirect ophthalmoscopy. Who to screen, and when to screen, depends on many factors, including the quality of the neonatal care provided. Where care is suboptimal, bigger, more mature babies should be screened as they can also develop sight-threatening ROP.

Since ROP is not present at birth, but develops during the first few weeks of life, the first screening examination should take place no later than 30 days after birth. Follow-up screening is often needed, and may be done after the baby has been discharged from the neonatal unit. Each country must decide which screening criteria apply to their setting.

All babies who develop the sight-threatening stages of ROP must be treated urgently: within 48–72 hours.

Follow-up of all preterm babies is important, as they are at greater risk of other conditions which can lead to visual loss (pp. 62–64). These are more common if the baby had ROP, particularly if treatment was given. The commonest condition is refractive error, including myopia, which can be severe and develop before the age of 12 months. Strabismus and cerebral visual impairment are also more common than in children born at term.

## New developments

There have been several new and important developments. These include the recognition that care of preterm babies during the first hour after birth is extremely important (this has been called the ‘first golden hour’). Kangaroo care, where the baby is placed securely on the chest of their mother or father (see below), can also play an important role in keeping preterm babies stable. New imaging systems for ROP are likely to change the way screening is undertaken, and new treatments for ROP are also being investigated. All of these topics are discussed in more detail in this issue.

## What can eye care providers do?

Nurses, neonatologists, ophthalmologists and parents all play a vital role in reducing the risk of ROP. However, in many low- and middle-income countries, lack of awareness about ROP is an issue, as it is not yet included in many training curricula, including those for paediatricians and ophthalmologists. There is also lack of awareness among the general population.

Ophthalmologists can visit the neonatal unit in the hospital, or a unit nearby, to find out whether preterm babies are admitted and survive, and whether babies are being screened for ROP. If not, they could set up a service (after being adequately trained).^4^,^5^

**Kangaroo care**

Kangaroo care helps to recreate an ideal environment for preterm infants. The infant is placed against the skin on the chest of the mother or father and held in place with a wrap. This can start as soon as the baby is stable, even if they have a medical condition. It can be intermittent or continuous.

Kangaroo care helps to keep babies stable and warm, increases maternal breast milk production and encourages breast feeding. This improves weight gain and growth which lowers the risk of mortality; there is also a lower risk of infection.

Kangaroo care promotes bonding between parents and their child and can help to reduce parental depression.

Some neonatal units have a dedicated ward for kangaroo care. Parents and their babies go there after leaving intensive care and before they are ready to go home.

The World Health Organization (WHO) has produced a practical guide to kangaroo care which is available from this link: **http://tinyurl.com/kangarooMC**

Evidence about the effectiveness of kangaroo care to reduce mortality and morbidity in preterm infants is available from: **https://www.ncbi.nlm.nih.gov/pubmed/27552521**

**Figure:**
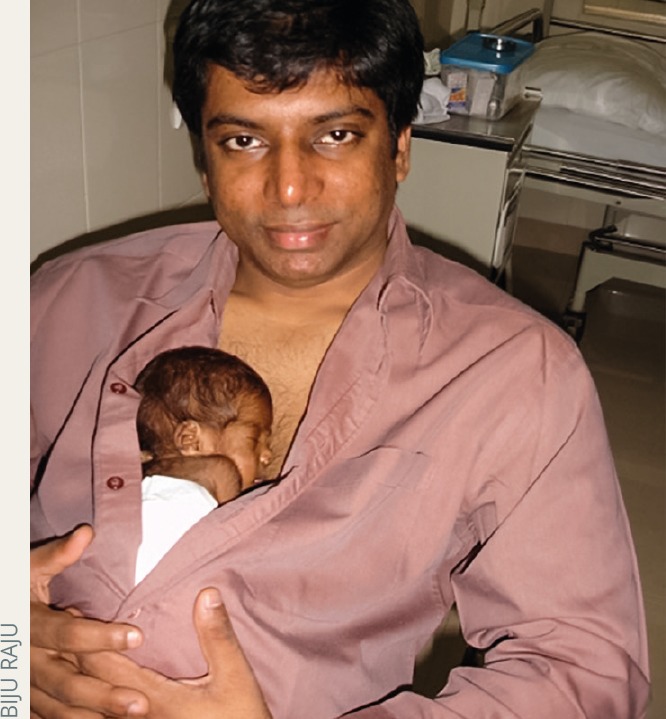
Ophthalmologist Biju Raju gave his son kangaroo care. Dr Raju screened (and treated) his son for ROP, despite initial protests from the neonatology team. INDIA

Ophthalmologists and optometrists can play an active role in following up infants and children who were born preterm to detect and manage refractive errors and other conditions, such as strabismus (pp. 62–64).

To improve awareness of ROP, eye care providers can distribute copies of relevant articles in this issue to colleagues, including obstetricians, midwives, neonatologists, neonatal nurses, paediatricians, ophthalmologists, and optometrists. The images are also helpful for educating parents.

Some infants with the advanced stages of ROP may retain a proportion of useful residual vision and will benefit from low vision services. Others may be completely blind. Since blindness of early onset can lead to developmental delay, these children should be referred for rehabilitation.

**Did you know?**

Sharing the articles in this issue can help to raise awareness of ROP. Copying and reuse of journal articles and images for such purposes is not only permitted, but encouraged. Online copies of all articles are available free of charge from **www.cehjournal.org** and high-resolution images are available (also free of charge) from **www.flickr.com/photos**

## Summary

A lot is now known about ROP in terms of the risk factors, which babies are most at risk and the natural history. In ROP there is only a very narrow time window in which to detect and treat babies who have the sight-threatening stages of ROP, i.e., within the first few weeks and months of life. Long-term follow up is essential. Many different people can play a role in preventing blindness and visual impairment from ROP and its long term complications Those providing low vision and rehabilitation services can help to improve children's future quality of life. Parents can play a critically important role at all stages of care.
